# Nucleus accumbens functional connectivity and circulating endocannabinoids levels in anorexia nervosa

**DOI:** 10.1192/j.eurpsy.2022.269

**Published:** 2022-09-01

**Authors:** R. Miranda-Olivos, I. Baenas, A. Pastor, A. Del Pino, E. Codina, I. Sánchez, A. Juaneda-Segui, S. Jimenez-Murcia, R. De La Torre, C. Soriano-Mas, F. Fernandez-Aranda

**Affiliations:** 1 Bellvitge University Hospital-IDIBELL, Psychiatry Department, Hospitalet del Llobregat, Spain; 2 Instituto Salud Carlos III, Ciber Fisiopatología Obesidad Y Nutrición (ciberobn), Madrid, Spain; 3 IMIM-Hospital del Mar Research Institute, Integrated Pharmacology And Systems Neurosciences, BCN, Spain; 4 Department of Public Health, Mental Health and Perinatal Nursing, School of Nursing, University Of Barcelona, Hospitalet, Spain; 5 CIBERSAM, Instituto Salud Carlos Iii, Madrid, Spain; 6 University of Barcelona, Department Of Clinical Sciences, LHospitalet, Spain; 7 Integrative Pharmacology and Systems Neuroscience, Hospital Del Mar Research Institute, BCN, Spain; 8 Universitat Pompeu Fabra, Department Of Experimental And Health Sciences, BCN, Spain; 9 Instituto Salud Carlos III, Ciber Salud Mental (cibersam), Madrid, Spain; 10 Universitat Autònoma de Barcelona, Departament De Psicobiologia I Metodologia De Les Ciències De La Salut, BCN, Spain; 11 School of Medicine and Health Sciences, University of Barcelona, Department Of Clinical Sciences, Barcelona -Campus Bellvitge, Spain

**Keywords:** nucleus accumbens, Endocannabinoids, resting-state functional connectivity, Anorexia nervosa

## Abstract

**Introduction:**

Neuroimaging findings have reported aberrant functional connectivity in brain regions involved reward system in individuals with anorexia nervosa (AN) altering hedonic processing over food. Likewise, endocannabinoids such as Anandamide (AEA) and 2-Arachidonoylglycerol (2-AG) have been involved in rewarding aspects of food intake.

**Objectives:**

To identify nucleus accumbens (NAcc) functional connectivity with whole-brain comparing between individuals with AN and controls. Furthermore, in a sub-study, to explore the interaction between NAcc functional connectivity and peripheral AEA and 2-AG levels.

**Methods:**

A total of 60 adult women (18 to 56 years of age) took part in the present study. Twenty-six individuals belonged to the AN group (BMI<18) and 34 to the HC group (BMI=18-24.99). All participants underwent functional magnetic resonance in resting-state, and blood samples were obtained in fasting.

**Results:**

Negative functional connectivity was observed in the AN group compared with the control group between the NAcc and the cerebellum (pFWE<.001), between the NAcc and the insula (pFWE<.001), between the NAcc and the supramarginal gyrus (pFWE=.019), and between the NAcc and the postcentral gyrus (pFWE=.010). Analyses exploring the association between NAcc functional connectivity and peripheral endocannabinoids levels displayed altered NAcc-cerebellum functional connectivity was negatively associated with peripheral 2-AG levels in the AN group (r= -.553; p=.011).

**Conclusions:**

Understanding the interaction between the reward system and peripheral endocannabinoids in patients with AN could contribute to better elucidate the pathophysiology of this disorder. Future studies will need to further investigate the clinical and therapeutic implications of these findings in patients with AN.

**Disclosure:**

No significant relationships.

